# Coaxial Electrospinning and Characterization of Core-Shell Structured Cellulose Nanocrystal Reinforced PMMA/PAN Composite Fibers

**DOI:** 10.3390/ma10060572

**Published:** 2017-05-24

**Authors:** Chao Li, Qingde Li, Xiaohui Ni, Guoxiang Liu, Wanli Cheng, Guangping Han

**Affiliations:** Key Laboratory of Bio-based Material Science and Technology (Ministry of Education), Northeast Forestry University, Harbin 150040, China; buxingzhelichao@163.com (C.L.); liqingde2017@hotmail.com (Q.L.); hnixiaohui@hotmail.com (X.N.); gxliunefu@hotmail.com (G.L.); nefucwl@nefu.edu.cn (W.C.)

**Keywords:** cellulose nanocrystals, coaxial electrospinning, core-shell structural nanofibers, thermal properties, fiber morphology

## Abstract

A modified coaxial electrospinning process was used to prepare composite nanofibrous mats from a poly(methyl methacrylate) (PMMA) solution with the addition of different cellulose nanocrystals (CNCs) as the sheath fluid and polyacrylonitrile (PAN) solution as the core fluid. This study investigated the conductivity of the as-spun solutions that increased significantly with increasing CNCs addition, which favors forming uniform fibers. This study discussed the effect of different CNCs addition on the morphology, thermal behavior, and the multilevel structure of the coaxial electrospun PMMA + CNCs/PAN composite nanofibers. A morphology analysis of the nanofibrous mats clearly demonstrated that the CNCs facilitated the production of the composite nanofibers with a core-shell structure. The diameter of the composite nanofibers decreased and the uniformity increased with increasing CNCs concentrations in the shell fluid. The composite nanofibrous mats had the maximum thermal decomposition temperature that was substantially higher than electrospun pure PMMA, PAN, as well as the core-shell PMMA/PAN nanocomposite. The BET (Brunauer, Emmett and Teller) formula results showed that the specific surface area of the CNCs reinforced core-shell composite significantly increased with increasing CNCs content. The specific surface area of the composite with 20% CNCs loading rose to 9.62 m^2^/g from 3.76 m^2^/g for the control. A dense porous structure was formed on the surface of the electrospun core-shell fibers.

## 1. Introduction

As nanotechnology advances and there is a general awareness of the significance of sustainability and the importance of renewable biological materials, there is a renewed interest in cellulose materials. Cellulose and its derivatives are the most abundant renewable organic materials produced in the biosphere, with an annual production estimated to be over 7.5 × 10^10^ tons [1]. Cellulose consists of crystalline and amorphous regions, from which highly ordered rod-like cellulose nanocrystals (CNCs) can be obtained via an acid hydrolysis [2,3]. CNCs have high mechanical properties, good thermal stability, nice biocompatibility, and ease of chemical modification [4], among other advantages. CNCs, as a reinforcement material, have enormous potential to improve the strength, modulus, and morphology of polymer matrix composites [5].

Many polymeric materials, such as polymethylmethacrylate (PMMA), polystyrene, and polyvinyl alcohol, have served as matrix materials because of their excellent consistency and low density. In the past few years, it has been common to use a polymer as a matrix to fabricate various composites reinforced with CNCs. These composite materials not only retain the original properties of the CNCs and the polymer but also have controllable composite performance improvement [6] and operations.

There are many methods for the preparation of a composite reinforced by CNCs. Electrospinning has advantages such as simplicity of operation, ease of control, and environmental protection. Using the electrospinning technique can produce composites with good mechanical stability [7], a high porosity structure [8], and excellent properties such as large specific surface area of the micro-nano-composite. Coaxial electrospinning has actualized the transformation of nanofibers from a single component to complex multi-level structures [9,10]. Core-shell structured nanofibers made by coaxial electrospinning have many applications in bio-medicine, optoelectronic devices, and absorption filtration. In the field of medicine, they can preserve unstable biological reagents or viruses effectively, to prevent the decomposition of unstable compounds. Core-shell structured nanofibers also serve as molecular drug slow release materials. Using an electrical conducting material as a core layer and an inert material as a shell layer, the core-shell nanofibers used in electrical cables can perfectly solve the electronic breakdown problem, which is a bottleneck in the development of microelectronic devices. Polyacrylonitrile (PAN) and PMMA have been used to prepare the composite electrical cable by uniaxial electrospinning [11], but this method has poor repeatability and many uncontrollable factors. It is easier to use the coaxial electrospinning process to prepare the core-shell nanofibers than to use a uniaxial electrospinning process.

Although the electrospinning process is simple and straightforward, many factors govern the mechanism for the formation of fibers, which involves a series of complex electro-fluid-mechanical stages, which affect the fiber diameter and morphology [12]. Thus, it remains challenging to control the production of finer polymer nanofibers with uniform diameter and structure [13,14]. In the process of coaxial electrospinning, technical parameters have a high impact on the formation of a coaxial jet, and furthermore, affect the performances of the resultant fiber. Low fluid viscosity makes it difficult for the core layer to follow the bending and shaking action of the shell fluid, and therefore perfect core-shell nanofibers with uniform structure are unattainable. When the shell fluid of a polymer matrix has a very high concentration, the process of coaxial electrospinning is also difficult for forming a good morphology of the nanofiber. For the preparation of coaxial composite nanofibers with superior morphology, a common approach is to reduce the concentration of the electrospinning solution to be as low as possible. However, a high enough chain-entangling density in the working solution is necessary to prevent capillary breakup and Rayleigh instability, which is the principal factor for producing thinner nanofibers with uniform structures. Unfortunately, the method of creating chain-entangling density often fails to produce high quality nanofibers. Other approaches to preparing thinner nanofibers include the manipulation of intrinsic spinning solution properties, the addition of additives such as salts and surfactants to the polymer solutions, and the control of process parameters such as the applied voltage and temperature [15]. Nevertheless, all these processes have resulted in some limitations for preparing nanofibers regarding nanofibers with unwanted components, limited thinning effects, and poor controllability. Thus, there is a need to develop a system to produce high-quality nanofibers in a controlled manner.

In this work, we used PMMA solutions with different CNCs contents as sheath fluids and PAN solution as the core fluid for preparing core-shell structured composite nanofibrous mats by a modified coaxial electrospinning process. The objectives of this study were to examine the morphology, thermal behavior, and the multilevel structure of the coaxial electrospun PMMA + CNCs/PAN composite nanofibers, and to further clarify the mechanism of CNCs loading on coaxial electrospinning. It is expected to expand the application of CNCs to electrospun materials.

## 2. Experimental Methods

### 2.1. Materials

Polymethyl methacrylate (PMMA) particles (Mw = 7.5 × 10^4^) and cellulose (50 μm) were purchased from the Aladdin Chemistry Co. Ltd. (Shanghai, China, AR grade), while Spectrum Chemical Mfg. Corp. (Shanghai, China) supplied polyacrylonitrile (PAN, Mw = 7 × 10^4^, AR grade), and Kermel Corp. and Beijing Chemical Works provided analytical grade N,N-Dimethylformamide (DMF) and sulfuric acid (AR grade, 98 wt %) respectively. These materials were used as received without further purification.

### 2.2. Preparation of Cellulose Nanocrystals

Ten grams of cellulose was mixed with 100 mL of 64 wt % sulfuric acid aqueous solution and the mixture was stirred vigorously at 45 °C for 1 h. Then, 1 L deionized water was added to the mixture to stop the hydrolysis reaction. The suspension stood for 24 h at 4 °C and was then centrifuged at 12,000 rpm for 20 min (TG16-WS, Cence Co., Shanghai, China), so that the CNCs separated from the suspension. Then, distilled water was used to wash the CNCs and the CNCs were separated from the mixture by centrifugation. It was necessary to repeat the process three times to complete the washing and separation of CNCs. The precipitate was placed in dialysis tubes and dialyzed against distilled water for several days until the water pH reached a value of 7.0 [16]. The pelleted CNCs obtained after dialysis were dried using a freeze dryer (Scientz-10N, Xin Zhi Co., Ningbo, China) [17,18].

### 2.3. Preparation of the Spinning Solution

One typical process for preparing the spinning solution in this work was:

1. Dissolve the PAN powder in DMF under vigorous magnetic stirring at 50 °C for 24 h to obtain a 16 *w/v* % concentrated PAN solution.
2. Dissolve PMMA particles in DMF under vigorous magnetic stirring at 50 °C for 24 h to obtain a 22 wt % concentrated PMMA solution.
3. Add the CNCs, obtained as described above, to the PMMA solution and agitate for 12 h followed by ultrasonic dispersion for 20 min before electrospinning. The CNCs loading levels in respect to the PMMA weight were chosen at 0, 5, 10, 15, 20 wt %. For the simplicity of discussion, we use PMMA + *x* CNCs to designate the samples, where *x* (wt %) is the CNCs loading level.

### 2.4. Coaxial Electrospinning Set-Up

**Figure 1:**
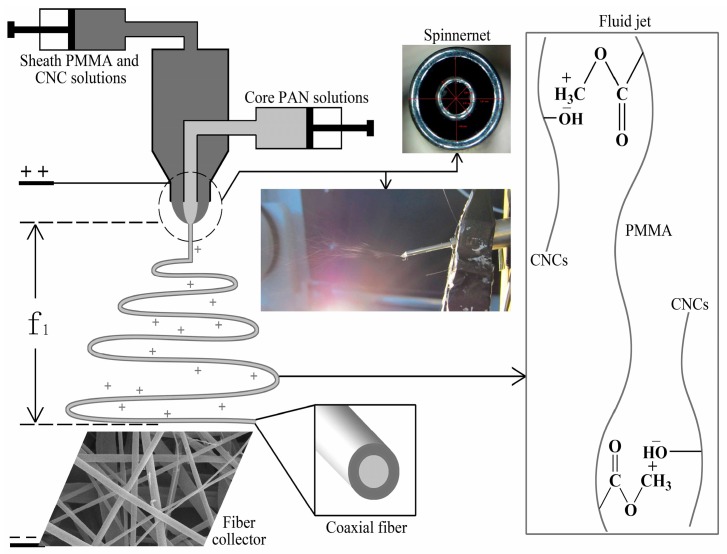
Schematic setup of coaxial electrospinning.

Figure 1 shows a schematic diagram of the coaxial electrospinning process. The inset of Figure 1 illustrates a typical fluid jet traveling process that is needed to have a small Taylor cone and a straight thinning jet. The pattern shows a bending and whipping instability region with loops of increasing size, and these are similar to the single-fluid electrospinning profiles [19]. In the concentric spinneret, the sheath fluid of the PMMA + *x* CNCs and the core fluid of PAN join from different syringe pumps. The core fluid and sheath fluid would not mix with each other in a very short time due to the lower diffusion coefficients of the two kinds of electrospinning liquid. At this time, the core fluid and sheath fluid formed a stable polymer micro-fluid with a high-speed jet under the influence of the electric field force. Under high-frequency tension, the shearing force generated at the interface between the core layer and the shell layer, and it made the core fluid take an axial movement. The coaxial electrospun composite nanofibers formed after solvent evaporation and solute solidification.

This study employed electrospinning (Yong Kang Le Ye Co., Beijing, China) with a 10 mL syringe (Zhi Yu Co., Shanghai, China) connected to a syringe pump to control the flows of the different solutions. It fed the sheath solution of PMMA + *x* CNCs and the core solution of PAN separately to each 10 mL syringe. The coaxial needles were obtained using an inner needle with 0.51 mm inner diameter (ID) and 0.81 mm outer diameter (OD). The outer needle had 1.46 mm ID and 1.81 mm OD. The sheath syringe pump supplied a steady flow of 0.02 mL/min of the sheath solution while the core syringe pump [20] fed a consistent flow of 0.034 mL/min of the core solution to the tip of the needle. A cylinder collector that rotated at a speed of 80 rpm was located at different distances from the needle tip to test the distances required for drying the fibers before hitting the collector. A rectangular piece of aluminum foil (200 mm in width) was covered on the cylinder to collect the nonwoven electrospun nanofibers. After some initial optimization experiments, it was determined to set the electrostatic field at 15 kV and collect the nanofibers on the aluminum foil at a distance of 20 cm from the needle tip. We placed a hygrothermograph inside the electrospinning chamber to monitor the relative humidity and temperature and made efforts to maintain the chamber at 22% and 25 °C, respectively.

### 2.5. Characterization of Spinning Solutions

A transmission electron microscope (TEM, Hitachi-7650, Hitachi, Tokyo, Japan) was used to examine the morphologies of the CNCs. We needed to dilute the aqueous CNCs suspensions to 0.1 wt % for analysis. The diluted suspensions were treated with an ultrasonic bath prior to the TEM analysis. A droplet of the diluted suspension was negatively stained with 5 mL of 2 wt % phosphotungstic acid for about 2 min to enhance the contrast of the TEM images. The mixture was then immediately deposited on the surface of a 400-mesh carbon-coated copper grid for examination under an accelerating voltage of 100 kV. The particle dimensions were calculated from the TEM images using the Nanometer software (Fudan University, Shanghai, China). The study randomly selected 60 particles to measure the particle dimensions from several TEM images of each sample.

The study also used a conductivity meter (DDSJ-318, Lei Ci Co., Shanghai, China) to characterize the conductivity of the PMMA + *x* CNCs and PAN solutions at room temperature. We also used a digital rotational viscometer (NDJ-5S, Wei De Co., Ningbo, China), and a surface tension meter (ZL-20, Ai Ji Co., Zibo, China) to determine the viscosity and surface tension, respectively. All measurements were carried out in triplicate.

### 2.6. Evaluation of Coaxial Electrospun Composite Nanofibers

After forming composite nanofibrous mats on the aluminum foils, a scanning electron microscope (SEM, QUANTA-200, FEI, Hillsboro, OR, USA) took microphotographs of each sample. We needed to trim and cut the mats into samples before coating with a layer of gold-palladium in vacuum followed by SEM examination at an accelerating voltage of 12.5 kV. Then, the Nanometer software analyzed and measured at least 80 nanofiber samples to determine the diameter and distribution of the electrospun nanofibers from the SEM images and also calculated the average fiber diameter.

**Figure 2:**
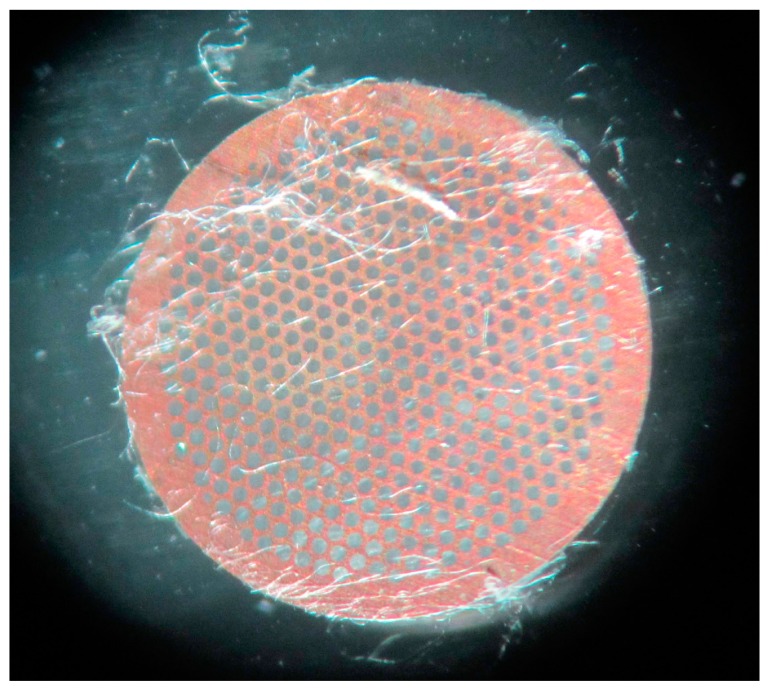
Coaxial composite nanofibrous mat spun on the microgrid.

We used a transmission electron microscope to examine the core-shell structure of an individual composite nanofiber at an accelerating voltage of 200 kV. In the preparation of the samples, the nanofibrous mat was electrospun on a microgrid in advance without staining (Figure 2). During the TEM examination, the individual fiber showed non-stop vibration caused by the high voltage electron beam irradiation, which made it difficult to capture a clear image. Two pieces of Lacey supported films overlapped together to hold the target fibers steady to solve the problem of vibration.

Fourier transform infrared spectroscopy (FTIR, NICOLET 6700, Thermo Fisher Scientific, Agawam, MA, USA) was used to obtain the spectra of each sample to examine any changes in the chemical structure of the coaxial electrospun PMMA + *x* CNCs/PAN composite nanofibers. Each spectrum was acquired in transmittance mode on a Zn/Se ATR crystal cell by the accumulation of 64 scans with a resolution of 4 cm^−1^ and a spectral range of 4000–500 cm^−1^. Spectral outputs were recorded as a function of the wavenumber. Also, the study employed thermogravimetric analysis (TGA, TGA-209, Netzsch, Germany) to investigate the thermal decomposition of coaxial electrospun PMMA + *x* CNCs/PAN nanofibrous mats. Samples of 8–10 mg were heated from room temperature to 600 °C at a rate of 5 °C/min under a nitrogen atmosphere for the thermogravimetric analysis.

An Accelerated Surface Area and Porosimetry System (ASAP2020, Micromeritics, Norcross, GA, USA) was used to study the specific surface area and mesopore size distribution. The specific surface area was calculated using the BET Equation (1) [21].

(1) $$\frac{p/p_{0}}{V(1-p/p_{0})}=\frac{c-1}{V_{m}c}\times \frac{p}{p_{0}}+\frac{1}{V_{m}c}$$

where *p* is the balance pressure (mmHg), *p*_0_ is the saturated vapor pressure of nitrogen (mmHg), *V* is the adsorption volume, *V_m_* is the monolayer adsorption saturation capacity (g·g^−1^), and *c* is a constant related to the adsorption heat and condensing heat.

The measurement of the gas adsorption capacity at different pressures was under isothermal conditions to obtain the isothermal adsorption curve. Nitrogen served as an adsorbed gas at room temperature.

The wettability of the coaxial electrospun PMMA + *x* CNCs/PAN nanofibrous mats was characterized on a contact angle meter (OCA20, Dataphysics, Bad Vilbel, Germany) at ambient conditions. The contact angle was measured using a sessile drop method. A droplet of 5 µL volume was used. The contact angle values of the right side and the left side of the water droplet were both measured and averaged. All of the contact angle data were an average of five measurements at different locations on the surface.

## 3. Results and Discussion

### 3.1. The Morphology of CNCs

**Figure 3:**
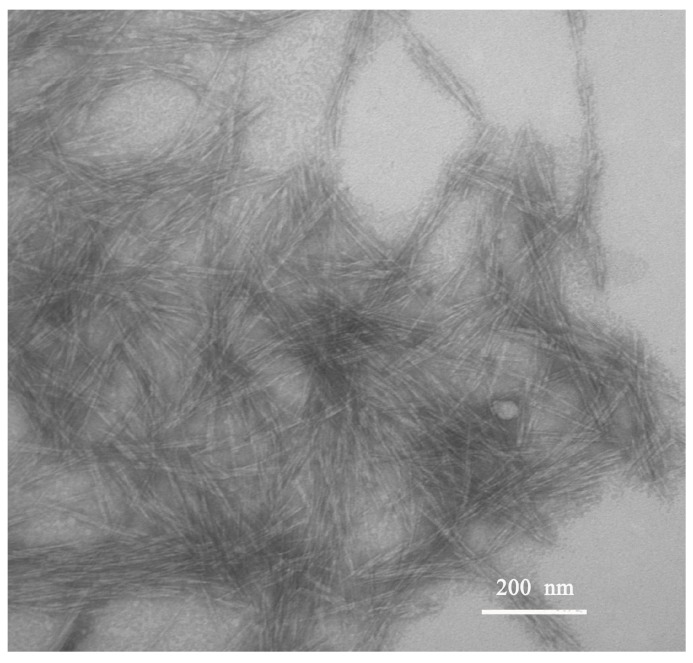
TEM photograph of CNCs.

Figure 3 displays the TEM images of the isolated CNCs solution. It reveals that the homogenized CNCs after treatment with 64 wt % H_2_SO_4_ dispersed well. The shape and size of the obtained CNCs were controlled and exhibited a rod-like structure with an average width of 13 ± 5 nm and a length of 100 ± 31 nm. The corresponding aspect ratio of the obtained CNCs was 7.7. Compared with the previously reported size of cellulose nanofibers produced from wood [22,23], the width of the obtained CNCs was similar, but their length was slightly shorter.

### 3.2. Properties of Spinning Solution

The physical and chemical properties of the electrospinning fluid are critical research parameters that directly affect the rheology and electrical properties of the electrospinning fluid, and further influence the morphology of electrospun fibers. Table 1 shows the conductivity, viscosity, and surface tension data of pure PAN, PMMA, and PMMA + CNCs suspensions with different concentrations of CNCs. The conductivity significantly increased with increasing CNCs loading, attributed to the high uronic acid and sulfate ester groups on the surface of CNCs obtained from the sulfuric acid hydrolysis process [24]. The presence of sulfonic groups improved the charge density of the fluid surface. Thus, the fluid exposed to a larger electric field force would help to reduce the fiber diameter and increase the sedimentary area of fibers [25]. The viscosity for the PMMA + CNCs suspensions was observed to increase with the incorporation of CNCs. CNCs and PMMA are both polar materials, and as expected they had excellent compatibility after their physical blending. The long chain of CNCs and PMMA would connect to each other, which improved the overall solution concentration to a certain extent, and therefore caused the increased viscosity. The surface tension of the PMMA + CNCs suspensions increased slightly compared with that of the PMMA solution. The study by Fong et al. [26] demonstrated that the use of low surface tension solvents resulted in a reduction in bead formation.

**Figure 4:**
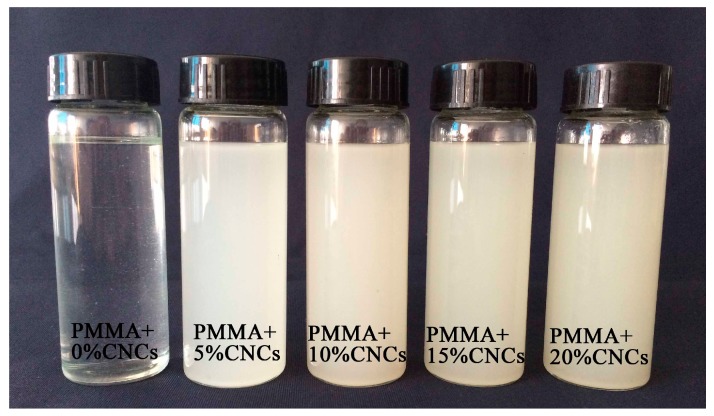
Photograph of pure PMMA solutions and PMMA + CNCs suspensions.

Figure 4 shows a photograph of pure PMMA solutions and PMMA + CNCs suspensions placed on a background paper. As shown in Figure 4, PMMA formed a clear solution in DMF. With the addition of CNCs to the system, the suspension transformed to an opalescent suspension. The suspensions with different CNCs loadings showed little precipitation after long retention times.

### 3.3. The Morphology of Coaxial Electrospun Composite Nanofibers

**Figure 5:**
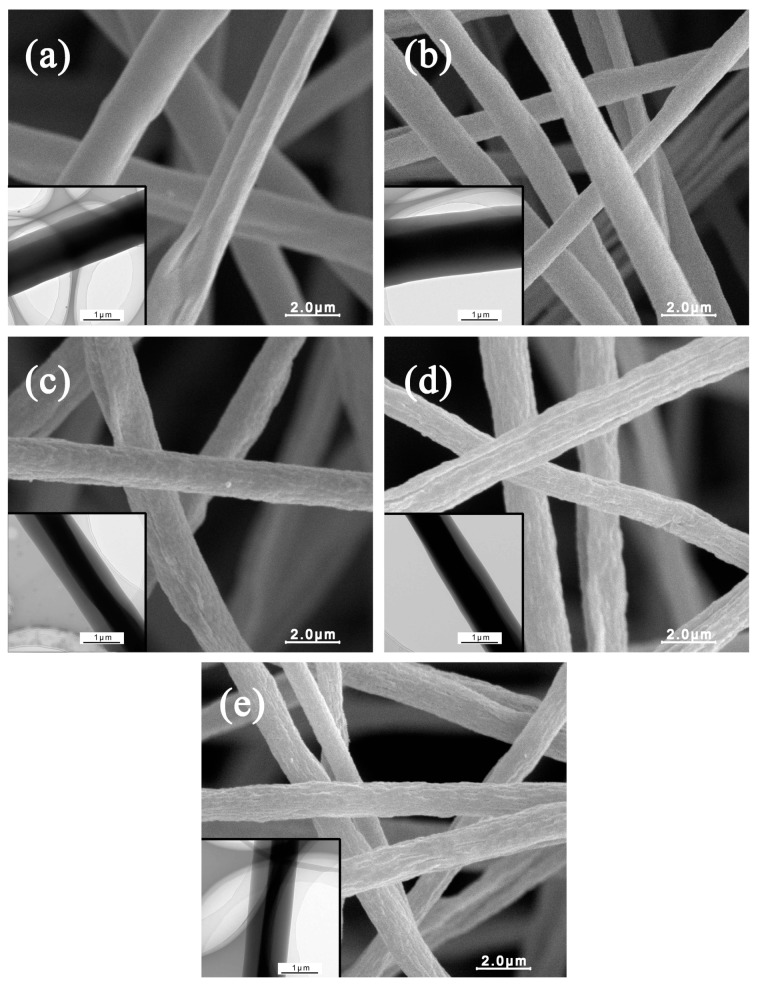
SEM and TEM images of coaxial composite fibers. (**a**) PMMA/PAN; (**b**) PMMA + 5% CNCs/PAN; (**c**) PMMA + 10% CNCs/PAN; (**d**) PMMA + 15% CNCs/PAN; (**e**) PMMA + 20% CNCs/PAN.

Figure 5 reveals the SEM and TEM images of coaxial electrospun composite fibers with different CNCs contents. It shows that the nanofibers without CNCs exhibited smooth surface structures. Figure 5c–e illustrates that with the increment of CNCs loading, the fiber surface was not smooth but had a convex and concave appearance, and the porosity of the surface increased gradually. A large number of convexes and micropores are shown on the fiber surface at 20% CNCs loading. The CNCs used were in crystal form when they were incorporated into the PMMA solutions. The presence of hydroxyl and sulfonic acid on the CNCs surface made it electronegative. The methyl on the long-chain molecule of PMMA was electropositive due to the electrical absorption (-I) of oxygen. Therefore, the electrostatic attraction formed between CNCs and PMMA made the stable shell fluid. Nevertheless, on the molecular level, CNCs are crystals consisting of many cellulose chains that cannot dissolve in the PMMA solutions. However, CNCs were able to disperse in the PMMA solutions homogeneously under the action of mixing and ultrasonic dispersion. In the spinning process, as the solvent evaporated, PMMA could attract some CNCs leading to a rough fiber surface. Another important reason is that the increased solution viscosity after adding the CNCs can also cause folding and uneven deposition on the surface of the electrospun fibers. The inset TEM images in Figure 5 illustrates the formation of the core-shell structure. It clearly shows that the color of the PMMA + CNCs shell is lighter than that of the core PAN. The shell color is uniform and stable, which indicated little CNCs aggregation in the shell [27].

**Figure 6:**
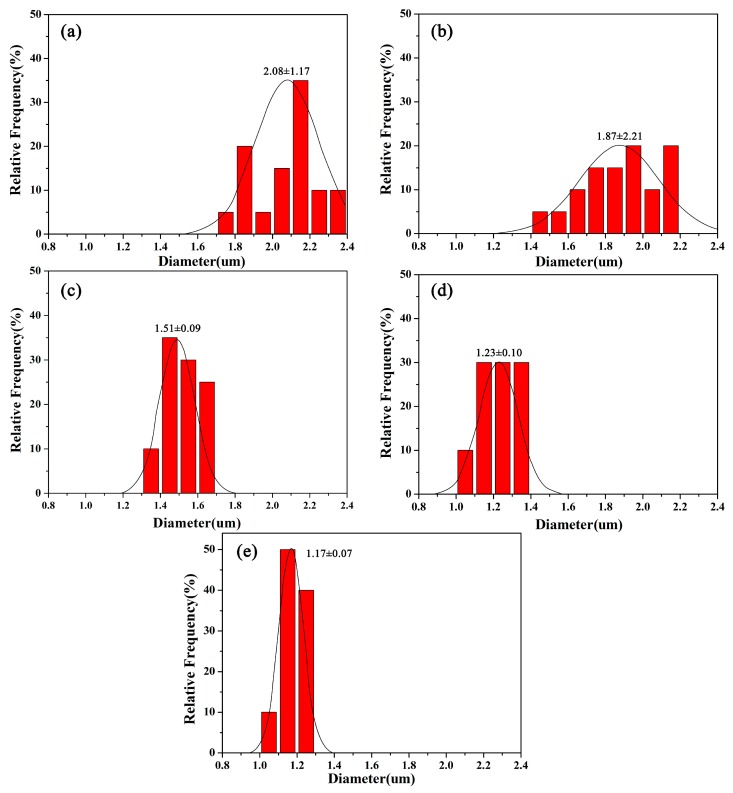
Diameter distribution of coaxial composite fibers. (**a**) PMMA/PAN; (**b**) PMMA + 5% CNCs/PAN; (**c**) PMMA + 10% CNCs/PAN; (**d**) PMMA + 15% CNCs/PAN; (**e**) PMMA + 20% CNCs/PAN.

Figure 6 displays the calculated average diameters and their deviations of electrospun nanofibers from different CNCs loadings. All the nanofibers exhibited uniform diameters without visible bead or bead-to-string structures. The average diameter of coaxial electrospun PMMA/PAN nanofibers was about 2.08 μm. The average diameter of nanofibers decreased with increasing CNCs loading. The average diameters of electrospun nanofibers were 1.87, 1.51, 1.23, and 1.17 μm for the fibers spun from the solution containing 5%, 10%, 15%, and 20% CNCs fibers, respectively.

The variations of viscosity, surface tension, and conductivity of the spinning solutions played decisive roles in determining the structure and diameter of the electrospun nanofibers [12,28]. The decrease of the fiber diameter and its distribution was due to the incorporation of CNCs. In the process of acid hydrolysis, the sulfonic groups (–SO_3_H^−^) partially displaced the hydroxyl groups on the surface of the CNCs [29], which resulted in the increased conductivity of the electrospinning liquid. Thus, the addition of CNCs produced smaller diameter nanofibers with uniform distribution [26], according to Equation [30]:

*D* ~ *Q_t_*^1/2^(*kγ*)^−1/6^ (2)

where *D* is the liquid jet diameter (μm), *Q_t_* is the inner and outer layer liquid volumetric flow (mL/h), *k* is the electrical conductivity of the solution (μs·cm^−1^), and *γ* is the surface tension of the solution (mN·m^−1^). As shown in Table 1, the surface tension changed little, but the conductivity increased significantly. From the equation, the increase in conductivity is inversely proportional to *D*, indicating the decreased fiber diameter.

The viscosity of the pure PMMA solution (396 MPa·s) was less than that of the pure PAN solution. After adding CNCs, the viscosity of the shell electrospinning fluid increased, leading to a higher density of its long chain molecule, which provided the necessary conditions to keep a consistent jet and Rayleigh instability. For the formation mechanism of the coaxial composite fiber, when the viscosity of the shell solution was higher than that of the core solution, it was beneficial to produce the shell fluid under high-frequency tension, which made the core fluid bending and swinging by the shear force from the direct-current high voltage electric field. The shell fluid serves as a process aid for the core fluid [31]. The uniform core-shell structure formed and the nanofibers ultimately deposited on the collecting board.

### 3.4. FTIR of Coaxial Electrospun Composite Nanofibers

**Figure 7:**
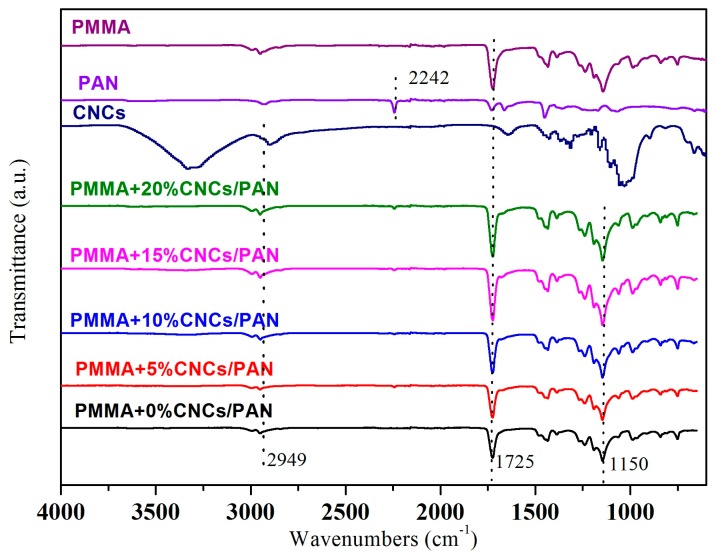
FTIR spectra of PMMA, PAN, CNCs, and coaxial composite nanofibers.

Figure 7 illustrates the FTIR spectra of the PMMA, PAN, CNCs, and coaxial electrospun composite nanofibers. For the pure PMMA nanofibers, the characteristic absorbance peaks around 1700 cm^−1^ are due to the stretching vibration of C=O. The band at 1150 cm^−1^ is due to the presence of C–O–C of the PMMA. The sharp and narrow band at 2242 cm^−1^ results from the stretching vibration of C≡N. For the original CNCs powders, the absorbance peaks in the range of 3600–3200 cm^−1^ are due to the stretching of the –OH groups of cellulose. The peaks around 2900–2800 cm^−1^ correspond to C–H stretching. The peaks observed at 1425 and 1315 cm^−1^ in the spectrum of the CNCs are due to the symmetric bending of –CH_2_ and the bending vibrations of the C–H and C–O groups of the rings in polysaccharides, respectively [32]. The stretching vibration at 1033 cm^−1^ was due to C–O at six- the membered carbocycle of the CNCs. For the coaxial composite nanofibers, a new peak appeared at 1033 cm^-1^ attributed to C–O of the CNCs and strengthened as the addition of CNCs increased. The peaks were enhanced at 2949 cm^−1^ and 1725 cm^−1^. The peak of the –OH group on the CNCs almost disappeared due to the small addition of CNCs to the polymer matrix of PMMA. The peak at 2242 cm^−1^ disappeared for the coaxial composite nanofibers because the shell solution enveloped the core solution. Thus, the C≡N in the core solution was not tested in the FTIR spectra. The FTIR spectra also demonstrated that the PMMA, PAN, and CNCs were physically blended. No chemical reaction occurred, and chemical bonds did not form during the coaxial electrospinning process.

### 3.5. Thermal Behavior of Coaxial Composite Nanofibers

**Figure 8:**
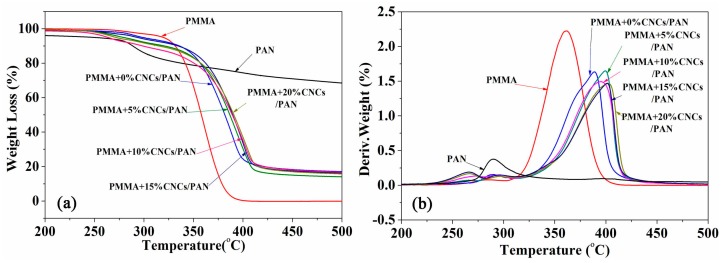
TG (**a**) and DTG (**b**) curves of the coaxial composite fibers.

Figure 8 shows the TG and DTG curves of the coaxial composite nanofibers. The initial decomposition temperatures of pure PMMA and PAN were 310 °C and 275 °C, respectively. There is a slight difference in the initial decomposition temperatures between the coaxial composite nanofibers and the electrospun nanofibers from pure PMMA or PAN. However, the DTG curves showed that the maximum decomposition rate of the coaxial composite nanofibers was far greater than that of any individual fiber components. As the CNCs content in PMMA + CNCs/PAN coaxial composite nanofibers increased, the maximum decomposition temperatures continued to rise. When 20 wt % CNCs were incorporated, the thermal decomposition temperature increased to 402.7 °C. This value was higher than the thermal decomposition temperature of pure PMMA, PAN, and the coaxial composite nanofibers without CNCs that were about 42.4 °C, 113.0 °C, and 13.9 °C, respectively.

The excellent thermal performances of the PMMA + CNCs/PAN coaxial composite nanofibers are due to two main reasons. The core-shell structure of the coaxial composite nanofibers is beneficial for their outstanding performance in heat transfer efficiency compared with the electrospun nanofibers from pure PMMA or PAN. The DTG curves indicated that the maximum pyrolysis temperature of the PMMA/PAN coaxial composite nanofibers was much higher than that of pure electrospun PMMA and PAN nanofibers. The pyrolysis rate of PMMA/PAN coaxial composite nanofibers was far less than that of pure PMMA nanofibers. The unique core-shell physical structure of coaxial composite nanofibers exhibited better thermal stability than the solid structure of electrospun nanofibers from any individual component, which demonstrated the complementary advantages in thermal effects for pure PMMA and PAN. Another important factor of the incorporation of CNCs was to provide excellent thermal performance. This thermal behavior resulted from a combination of the effect of hydrogen bond crosslinking between CNCs and the effect of mechanical percolation [33]. The improved thermal stability of PMMA + CNCs/PAN coaxial composite nanofibers could be because the CNCs interconnect with each other during electrospinning to create a rigidly interconnected network. Furthermore, the surface sulfonic acid group (−SO_3_H^−^) of CNCs could mingle with each other to form a physical crosslinking structure, which also increased the thermal stability.

### 3.6. The Specific Surface Area and Pore Size Distribution of Coaxial Composite Nanofibers

Figure 5 shows that the PMMA + CNCs/PAN coaxial electrospun composite nanofibers had more microvoids with convexes and concaves in the SEM images. A nitrogen adsorption method was used to determine the specific surface area and the surface pore structure. We used the BET (Brunauer, Emmett and Teller) specific surface area Equation (1) to calculate the specific surface areas of PMMA/PAN, PMMA + 5% CNCs/PAN, PMMA + 10% CNCs/PAN, PMMA + 15% CNCs/PAN, and PMMA + 20% CNCs/PAN coaxial composite fibers. The results showed that the surface areas of the coaxial composite fibers increased with increasing CNCs content. As the CNCs content increased from 0%, 5%, 10%, 15%, to 20%, the surface areas increased from 3.76, 5.18, 6.83, 8.37, to 9.62 m^2^/g, respectively.

**Figure 9:**
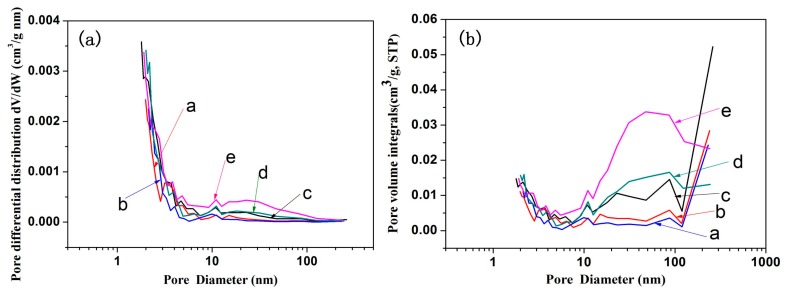
Pore size distribution curve (**a**) and pore volume integral curve (**b**) of coaxial composite fibers. (**a**) PMMA/PAN; (**b**) PMMA + 5% CNCs/PAN; (**c**) PMMA + 10% CNCs/PAN; (**d**) PMMA + 15% CNCs/PAN; (**e**) PMMA + 20% CNCs/PAN.

Figure 9 displays the pore size distribution curve and pore volume integral curve of the coaxial composite fibers. A large number of micropores and mesopores exist on the surface of the PMMA + CNCs/PAN coaxial composite nanofibers. The range of the pore size distribution increased, and the number of micropores increased with increasing CNCs loading. The reason for this phenomenon is that water molecules as a solvent would cause the non-water-soluble polymer and water to have a phase separation in the process of electrospinning, which would then produce a porous structure on the surface of the nanofibers [34]. Hydrophilic hydroxyl groups on the surface of the CNCs absorbed water molecules from the air, which prompted coaxial composite nanofibers to produce a porous structure in the electrospinning process. Another reason would be that the CNCs are not likely to be perfectly aligned with the flow direction, and those nanofibers could 'indent' the surface [35].

**Figure 10:**
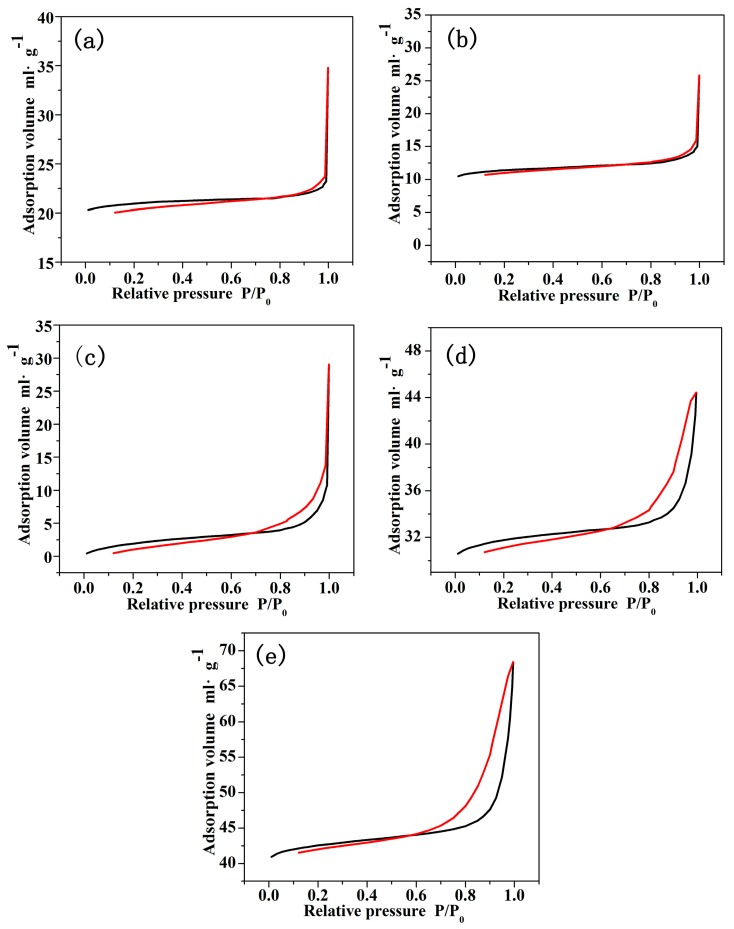
Adsorption-desorption curves of coaxial composite fibers. (**a**) PMMA/PAN; (**b**) PMMA + 5% CNCs/PAN; (**c**) PMMA + 10% CNCs/PAN; (**d**) PMMA + 15% CNCs/PAN; (**e**) PMMA + 20% CNCs/PAN.

Figure 10 shows the adsorption-desorption isotherms for the PMMA + CNCs/PAN coaxial composite nanofibers. Examination of Figure 10a,b indicates that the samples exhibited combined characteristics of type III curves according to the IUPAC (International Union of Pure and Applied Chemistry) classification. The curves of Figure 10c–e are hysteresis loops, belonging to IV curves. The type III curves indicate weak interactions of gas and solids on the nonporous or macroporous solid. Since a mesoporous solid generates type IV curves and the coaxial composite nanofibers are characterized with type IV curves, the coaxial composite nanofibers are more mesoporous. The mesopores on the nanofibers occur due to capillary condensation phenomenon under high relative pressures. Figure 10a,b shows that sharp growth took place when the relative pressure P/P_0_ reached 1.0, indicating that the material had a nonporous structure. The first half of the curves in Figure 10c–e showed a slow increase, while for the second half, the adsorption volume quickly increased with increasing relative pressure. Nitrogen adsorption-desorption isotherms appeared as hysteresis loops at the high relative pressure P/P_0_, which demonstrated that the capillary condensation and evaporation phenomenon occurred in the material and indirectly proved the presence of narrow pores and fissures in the coaxial composite nanofibers [36]. All of the data illustrated that the specific surface area of the coaxial composite nanofibers increased with the increase in CNCs loading. Nitrogen adsorption-desorption isotherms proved that the coaxial composite nanofibers containing CNCs gradually changed from ordinary nonporous or macroporous to porous fibers containing a higher proportion of mesopores. The adsorption volume significantly increased with the increase of CNCs loading. The results of the increased specific surface area and porous structure provide approaches for the application of coaxial composite nanofibers in drug release and ion adsorption [37].

### 3.7. Wettability of Coaxial Electrospun Composite Membrane

The micro-surface roughness and surface free energy of a material are the main factors affecting its surface wettability. The increase of micro surface roughness improves the material’s hydrophobic properties. In the micro-environment, the liquid dropping on a solid surface cannot completely fill the concave of rough solid surfaces. Air exists between the liquid droplets and the solid concave. Since the contact angle of air and water molecules is about 180°, the rougher the micro surface is, the more air is sealed off, and the less opportunities for the solid surface to contact with water, the more hydrophobic it will be. For the electrospun membrane, the diameter of the fiber determines the surface roughness of the thin film. The larger the fiber diameter is, the rougher the film is, and the more hydrophobic the material will be. The smaller the fiber diameter is, the less rough the film surface is, and thus the hydrophobicity of the material will reduce.

**Figure 11:**
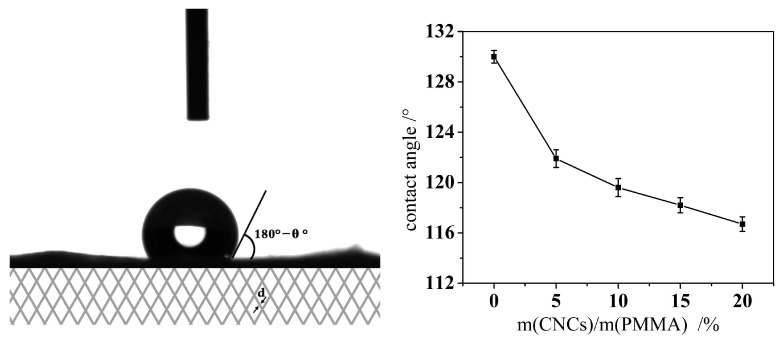
Photograph of a water droplet on a solid surface and the contact angle values of the coaxial composite membrane.

The surface free energy is another major factor that affects the hydrophobicity of the material. The –OH groups on the CNCs have higher free energy because of their hydrophilic nature. The –COO– ester groups of the PMMA molecules are hydrophobic and lipophilic groups. Figure 11 shows a photograph of a water droplet on the solid surface and the contact angle values of the coaxial electrospun composite membrane. The contact angle of the coaxial electrospun composite membrane without CNCs was 130°, indicating its hydrophobic features. This hydrophobicity of the membrane relates to the hydrophobic properties of the ester groups of the PMMA molecule and the higher surface roughness caused by the larger diameter of the nanofibers. The contact angle value decreased from 130° to 121.9° when the CNCs content increased from 0% to 5%. As the CNCs content further increased, the contact angle reduced to 116.7° because the –OH hydroxyl of the CNCs molecule is hydrophilic. As the CNCs content increased, the number of hydroxyl groups (–OH) in the membrane expanded as did its hydrophilicity, which led to a smaller contact angle [6].

## 4. Conclusions

In this study, we fabricated, investigated, and discussed PMMA/PAN electrospun coaxial composite nanofibers with different CNCs loading levels in the shell solution of PMMA regarding the morphology, thermal performance, specific surface area, and surface structure features of the material. The main conclusion of this study is that the coaxial electrospun nanofibers with CNCs have some unique characteristics and properties, which could have many applications such as drug release and ion absorption. The major conclusions are:

1. The fiber diameter decreased from 2 μm to 1.17 μm when the CNCs level increased from 0% to 20%, while its distribution narrowed.
2. The thermal stability of coaxial electrospun composite nanofibers was significantly enhanced.
3. CNCs were physically mixed with PMMA and probably distributed on the shell surface of the coaxial composite nanofibers according to the FTIR analysis.
4. The specific surface area of the core-shell composite significantly increased with increasing CNCs content.
5. The surface structure of the fibers gradually changed from a non-hole to porous surface that contained a higher proportion of mesoporous material.
6. The hydrophobic properties of the coaxial electrospun composite membrane reduced due to the presence of –OH hydroxyl groups on the CNCs.

## Figures and Tables

**Table 1:** Properties of the as-spun solutions ^a^.

Solution	Conductivity (μS·cm^−1^)	Viscosity (mPa·s)	Surface Tension (mN·m^−1^)
PAN	30.4 ± 2.1	500 ± 8	29.6 ± 7
PMMA	2.06 ± 0.11	396 ± 5	23.4 ± 4
PMMA + 5% CNCs	7.55 ± 0.33	612 ± 6	24.9 ± 4
PMMA + 10% CNCs	15.8 ± 0.71	704 ± 9	25.8 ± 5
PMMA + 15% CNCs	29.2 ± 1.29	840 ± 11	26.2 ± 7
PMMA + 20% CNCs	33.4 ± 1.33	888 ± 12	26.6 ± 7

^a^ Date are given as the mean value ± standard deviation at 25 °C.
